# Fragile X premutation carrier screening in Pakistani preconception women in primary care consultation

**DOI:** 10.1186/s12905-022-01632-1

**Published:** 2022-03-04

**Authors:** Neelam Meraj, Muhammad Yasin, Zia Ur Rehman, Haleema Tahir, Humaira Jadoon, Niamat Khan, Rabia Shahid, Maria Zubair, Irba Zulfiqar, Musarrat Jabeen, Shahzadi Neelam, Abdul Hameed, Shamim Saleha

**Affiliations:** 1 Department of Biotechnology and Genetic Engineering, Kohat University of Science and Technology (KUST), Kohat, 26000 Khyber Pakhtunkhwa Pakistan; 2 Department of Obstetrics and Gynecology, Ayub Medical Institute, Abbottabad, 22010 Khyber Pakhtunkhwa Pakistan; 3 Department of Obstetrics and Gynecology, Liaqat Memorial Hospital, KIMS, Kohat, 26000 Khyber Pakhtunkhwa Pakistan; 4 Department of Obstetrics and Gynecology, Qazi Ahmed Medical Complex, Nowshera, 24100 Khyber Pakhtunkhwa Pakistan; 5 Institute of Biomedical and Genetic Engineering (IBGE), Islamabad, 44000 Pakistan

**Keywords:** *FMR1*, PM carrier screening, Fragile X associated disorders, Risk groups, Pakistani preconception women

## Abstract

**Purpose:**

Women of reproductive age who carry fragile X premutation (PM) alleles have 56 to 200 CGG repeats in the 5′-untranslated region of *FMR1* gene are at increased risk for producing children with intellectual disabilities (ID) or autism spectrum disorders (ASD) due to expansion of PM alleles to full mutation alleles (> 200 repeats) during maternal transmission.

**Methods:**

In present study fragile X PM carrier screening was performed in total 808 women who were consulting primary health care centers for preconception care in Khyber Pakhtunkhwa region of Pakistan between April, 2018 and December, 2020. Polymerase chain reaction (PCR) was performed for detection of PM carrier women and the CGG repeats number was confirmed by Southern blotting and capillary electrophoresis.

**Results:**

The prevalence rate for PM carriers among preconception women was found to be 0.7% that was contributed by 0.5% women in risk group (RG1) with family history of ID and 0.2% in risk group 2 (RG2) with family history of ASD. PM carrier women had at least one affected child or sibling. In addition, the preconception women with *FMR1* PM alleles were found to be at increased risk for primary ovary insufficiency (RG1: *P* = 0.0265, RG2: *P* = 0.0389), postpartum depression (RG1: *P* = 0.0240, RG2: *P* = 0.0501) and neuropsychiatric disorders (RG1: *P* = 0.0389, RG2: *P* = 0.0432).

**Conclusions:**

Current study provides first evidence of fragile X PM carrier screening in Pakistani preconception women in primary care consultation. Findings of current study may help to improve preconception care and to reduce burden of fragile X associated disorders in our population.

## Introduction

Fragile X syndrome (FXS) is a rare neurodevelopmental disorder (MIM # 300,624) that affects approximately 1 in 4000 males and 1 in 6000–8000 females [1]. FXS is characterized by a wide range of inherited intellectual disabilities and autism spectrum disorders in children [2]. FXS is caused by the cysteine-guanine-guanine (CGG) repeat expansion mutations in 5′-untranslated region (UTR) of the *FMR1* gene on chromosome X. The CGG repeat expansion mutations cause gene methylation and in turn inactivation of the *FMR1* gene [3]. Importantly, the number of CGG repeats size is not constant among individuals. There are four allelic forms of the *FMR1 *gene based on CGG repeat length. They are known as normal alleles with less than 45 repeats, intermediate alleles with 45–54 repeats, permutation (PM) alleles with 55–200 repeats and full mutation (FM) alleles greater than 200 repeats [4]. Individuals with PM alleles are considered carriers and those with FM alleles are referred affected and exhibit FXS clinical phenotypes [5].

Notably, about 1 in 150–300 women carry PM alleles, however, PM carrier frequency may vary among women from different ethnic groups [6]. PM carrier women usually are at increased risk to develop fragile X associated primary ovarian insufficiency (FXPOI) [7] and fragile X associated diminished ovarian reserve (FXDOR) [8] in reproductive age as well as fragile X associated tremor/ataxia syndrome (FXTAS) in late age [9]. Women of reproductive age diagnosed with FXPOI may have symptoms of low minerals bone density, osteoporosis, bone fractures [10, 11] and fibromyalgia [12] PM carrier women may experience obstetric and perinatal difficulties like late pregnancy bleeding [13] and vestibular issues such as dizziness, spinning and not able to balance body [14]. PM carrier women may suffer from hypertension, central pain sensitivity syndrome, sleep problems, restless legs syndrome, migraine and gait issues [15]. Depression, anxiety and attention problems were more commonly observed psychiatric features in PM carriers [16]. These reported clinical features are also observed to be prevalent among non-PM carrier women, but the case of PM carrier women is more critical as they are at increased risk of producing affected children with FXS due to maternal PM alleles expansion to FM alleles during transmission [17].

Population based studies conducted in different countries have detected a significant number of PM carrier women, who transmitted PM alleles to fetus and delivered children with FXS [18, 19]. These studies strongly suggest counseling and screening of preconception women in primary care consultation for detection of PM carrier status that may be of paramount importance [8, 20]. In addition, the American College of Medical Genetics recommends *FMR1* PM carrier screening for preconception women with a family history of fragile X associated disorders (FXD) under a clinical research protocol. According to guidelines of ACOG, Polymerase chain reaction (PCR) and Southern blotting are the most preferred molecular diagnostic methods for detection of *FMR1* PM carrier status of preconception women [21, 22].

Here, we report first study for fragile X PM carrier screening among Pakistani preconception women of reproductive age in primary care consultation and also determined health risks associated with the *FMR1* PM carrier status of women.

## Materials and methods

### Study subjects

**Fig. 1:**
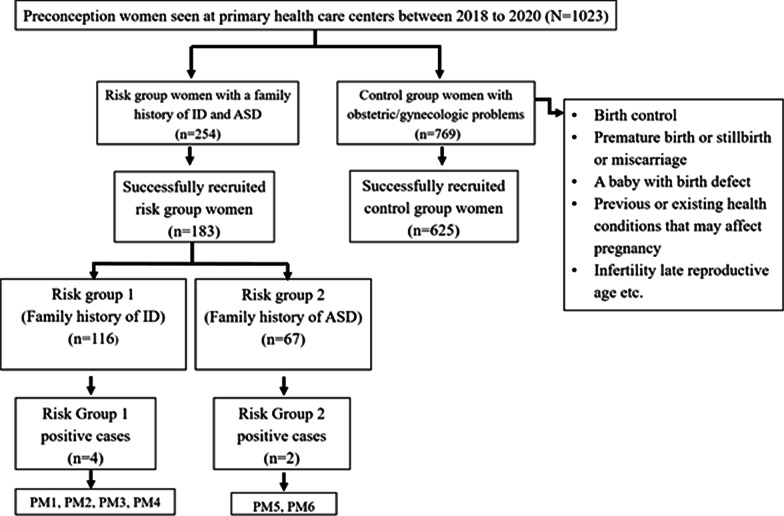
Flow diagram showing recruitment process and *FMR1* PM screening results

The approval of present study was obtained from the Ethical Committee and Advanced Studies Research Board (ASRB) of Kohat University of Science and Technology (KUST), Kohat, Khyber Pakhtunkhwa, Pakistan. The women of reproductive age who were consulting primary health care centers in Khyber Pakhtunkhwa region of Pakistan between April-2018 and December-2020 for preconception care. Before recruitment informed written consent was obtained from each participating woman. Women who fulfilled the criteria as described by ACOG for fragile X carrier screening of preconception women were recruited at primary health care centers for this study as shown in Fig. 1. Preconception women with a family history of either intellectual disability (ID) or autism spectrum disorder (ASD) were recruited in risk group 1 (RG1) and risk group 2 (RG2) respectively. To determine prevalence rate of *FMR1* PM alleles, women in preconception care with obstetric/gynecologic problems but had no family history of FXD and provided consent for participation in study were included in control group. A significant number of women with or without family history of FXD did not provide written informed consent or did not want to be counseled or couldn’t be counseled for participation in this study were excluded. Information on demographics, family medical history, health status, clinical investigations were collected from recruited women with help of obstetricians and gynecologists at primary health care centers.

### Fragile X carrier screening

The standard phenol–chloroform method was used to extract genomic DNA from peripheral blood of women participating in study. Polymerase Chain Reaction (PCR) was used as initial screening method for detection of fragile X carrier women. For amplification of *FMR1* alleles in the first step PCR, primers forward 5′-TCAGCTCCGTTTCGGTTTC-3′ and reverse 5′-CCTTGTAGAAAGCGCCATTG-3′ were designed. PCR amplifications were performed in a 25 μl reaction containing 2X DreamTaq Green PCR master mix (Thermo Scientific), 0.5 μM of each primer, 100 ng/μL of template DNA and 3% DMSO and 2.5 M Betaine. The cycling conditions for PCR were initial denaturation for 5 min at 95 °C, followed by 30 cycles of denaturation for 30 s at 95 °C, annealing for 30 s at 59 °C, extension for 30 s at 72 °C, and a final extension for 8 min at 72 °C. In second step PCR, DNA of women amplified as single bands in first step PCR were subsequently analyzed by using primers (c primer: 5′-GCTCAGCTCCGTTTCGGTTTCACTTCCGGT-3′ and CGG-chimeric primer:5′-AGCGTCTACTGTCTCGGCACTTGCCCGCCGCCGCCG-3′ for the random amplification of CGG repeats by optimizing PCR amplification conditions as described previously [23]. Furthermore, the CGG repeats number in *FMR1* PM alleles in carrier women and in their family members were determined by Southern blotting and capillary electrophoresis and for this purpose services of commercial diagnostic laboratories in Islamabad were utilized.

### Statistical analysis

The characteristics of the participating preconception women were described or summarized using the SPSS 21.0., software. To compare qualitative variables, the chi-square statistic was used and a *P* value < 0.05 was considered significant to find potential risk factors for *FMR1* PM carrier women.

## Results

In total 808 women screened for *FMR1* PM, majority of participating preconception women were in control group with no family history of FXD (77.35%). However, a substantial number of preconception women were in RG1 and RG2 with family history of either ID (14.35%) or ASD (8.3%) respectively (Fig. 1). Table 1 presents the frequencies of affected family members of recruited risk groups preconception women, however, the preconception women in RG1 and RG2 had at least one child or sibling affected with either ID or ASD respectively. The prevalence rate for PM carriers among preconception women was found to be 0.7% that was contributed by 0.5% women in RG1 and 0.2% in RG2 who were detected carries for PM alleles. However, PM alleles were not detected in any woman from control group. In addition, 21 (2.3%) women in RG1 and about 1.1% in RG2 were found to carry intermediate alleles. In addition, PM and FM alleles were also detected in family members of PM carrier women. Almost all PM carriers had low anti-müllerian hormone (AMH) levels (< 1 ng/mL) and high follicle stimulating hormone (FSH) levels (25.8 ≥ IU/L) as shown in Table 2 and Table 3. Additional characteristics of PM carrier women are summarized in Table 3 and health risks associated with PM carriers are shown in Table 4. Majority of PM carrier women (66.7%) were less than 33 years of age, suffered from irregular menstruation (83.3%) and hot splashes/ night sweats (66%). PM carriers’ women had a significant increased risk of developing FXPOI (RG1: *P* = 0.0265 and RG2: *P* = 0.0389). PM carrier women less frequently experienced obstetric and perinatal difficulties such as antepartum hemorrhage (33%), whereas more obviously experienced early onset osteoporosis (83.3%), however, no significant differences were found in PM carrier and non-PM carrier women with respect to these factors as shown in Table 4. Postpartum depression was more prevalent (83.3%) among PM carrier women and they were found at significantly increased risk for this health problem (RG1: *P* = 0.0240 and RG2 *P* = 0.0501). Although, a substantial number of PM carrier women suffered from hypertension (66%) and migraine (50%), however, PM carrier women were not found at increased risk for these health conditions (RG1: *P* = 0.1436 and RG2 *P* = 0.5384). Interestingly, all PM carrier women had normal intelligence quotient (IQ) levels. In addition, the most common neuropsychiatric features present in majority of PM carrier women were anxiety (83.3%), sleep disturbance (83.3%), aggression (83.3%), difficulty in concentrating (66%), hyperreactivity (66%), and language issues (50%). Importantly, PM carrier women were found at significantly increased risk for neuropsychiatric disorders (RG1: *P* = 0.0389 and RG2: *P* = 0.0432).

**Table 1:** Frequencies of affected family members of recruited risk groups preconception women

Affected family members	No. of affected individuals	Risk group 1 (%)	Risk group 2 (%)
Offspring	1	32	29
	2	41	46
	3	20	13
Sibling	1	1	3
	2	3	4
	3	3	5

**Table 2:** AMH and FSH levels in preconception women with *FMR1* PM

Hormonal levels	*FMR1* PM carriers					
	PM1	PM2	PM3	PM4	PM5	PM6
AMH level (ng/mL)	0.42	0.71	0.53	0.91	0.42	0.52
FSH level (IU/L)	37.21	28.1	38.3	25.8	33.3	42.3

**Table 3:** Characteristics of preconception women with *FMR1* PM Alleles

Characteristics	*FMR1* PM CARRIERS						Total (%)
	PM1	PM2	PM3	PM4	PM5	PM6	
Ages in years	25	29	30	32	35	37	
CGG repeats’ number	61	72	59	71	64	58	
Primary ovarian insufficiency symptoms							
Irregular menstruation	**+**	**+**	_	**+**	**+**	**+**	83.3
Skipped menstruation	_	**+**	**+**	_	_	_	33
Subfertility	_	_	_	_	_	**+**	16.6
Hot splashes and night sweats	_	_	**+**	**+**	**+**	**+**	66
Low AMH level	**+**	**+**	**+**	**+**	**+**	**+**	100
High FSH level	**+**	**+**	**+**	**+**	**+**	**+**	100
Difficulty getting pregnant	_	_	_	**+**	_	_	16.6
Obstetric and perinatal difficulties							
Antepartum hemorrhage	_	**+**	_	_	**+**	_	33
Obstructed labor	_	_	_	_	**+**	_	16.6
Pre-eclampsia	_	_	**+**	_	_	_	16.6
Hypoestrogenism conditions							
Lower bone mineral density	**+**	_	_	_	**+**	_	33
Early onset osteoporosis	**+**	**+**	**+**	**+**	**+**	_	83.3
Bone fractures	_	**+**	_	**+**	_	_	33
Postpartum depression	**+**	_	**+**	**+**	**+**	**+**	83.3
Medical conditions							
Hypertension	**+**	_	**+**	**+**	**+**	_	66
Migraine	**+**	_	_	**+**	**+**	_	50
Diabetes	_	_	**+**	_	_	_	16.6
Obesity	**+**	_	_	**+**	_	_	33
Neuropsychiatric features							
Language issues	_	**+**	**+**	_	_	**+**	50
Memory loss	_	**+**	_	_	_	**+**	33
Trouble performing daily activities	**+**	**+**	_	_	_	_	33
Normal IQ level	**+**	**+**	**+**	**+**	**+**	**+**	100
Difficulty in concentrating	**+**	**+**	**+**	_	_	**+**	66
Anxiety	**+**	**+**	**+**	_	**+**	**+**	83.3
Attention deficit disorder	**+**	_	_	_	**+**	_	33
Sleep disturbance	**+**	**+**	**+**	_	**+**	**+**	83.3
Aggression	**+**	**+**	**+**	_	**+**	**+**	83.3
Reasoning (irritability)	_	**+**	_	_	**+**	_	33
Hyperreactivity	**+**	**+**	**+**	_	**+**	_	66

**Table 4:** Health risks associated with PM carrier preconception women

Risk factors	Risk group 1 (N = 116)		p-value	Risk group 2 (N = 67)		p-value
	Positive (n = 4)	Negative (n = 112)		Positive (n = 2)	Negative (n = 65)	
Ovarian insufficiency symptoms						
Yes	4	25	*0.0265	2	10	*0.0389
No	0	87		0	55	
Obstetric and perinatal difficulties						
Yes	2	27	0.2607	1	20	0.5725
No	2	85		1	45	
Hypoestrogenism conditions						
Yes	4	47	0.0996	1	18	0.5043
No	0	65		1	47	
Postpartum depression						
Yes	3	19	*0.0240	2	11	*0.0501
No	1	93		0	54	
Medical conditions						
Yes	3	39	0.1436	1	19	0.5384
No	1	73		1	46	
Neuropsychiatric features						
Yes	3	23	*0.0389	2	10	*0.0432
No	1	89		0	55	

*Significant

## Discussion

Expansion of CGG repeats > 200 in the 5′-UTR of the *FMR1* gene on the X chromosome and subsequent epigenetic modifications are the most common cause of inherited ID and monogenic cause of ASD usually in males [2, 6, 24]. Importantly, fragile X PM carrier women with < 200 repeats are at increased risk for producing affected children due to expansion of repeats to FM that occurs almost only in transmission from mother to children [25]. In addition, the family history of FXD of women increases significantly risk of having children with FXS [26, 27]. Fragile X PM carrier status is essentially silent in women of reproductive age unless they develop POI, thus family history of FXD may lead clinicians to diagnose PM carrier women in reproductive age in populations [28]. Therefore, ACOG has recommended *FMR1* carrier screening of preconception women with a family history of FXD to determine PM carrier status of women [21, 22]. Primary health care is recognized as a setting where direct approach to preconception women for screening genetic disorders like FXS may be possible. Diagnosis of fragile X PM carrier status of preconception women may help in making reproductive decisions and family planning [26].

According to our knowledge, this is a first study in which fragile X PM carrier screening was performed in women who were consulting obstetricians and gynecologists for preconception care at primary health care centers in Khyber Pakhtunkhwa region of Pakistan. An important aspect of this study is participation of obstetricians and gynecologists’ other clinicians that make feasible this study in clinical setting as recommended by guidelines of the ACOG [21, 22]. In consistent with our study, fragile X carrier screening was preferentially offered to women before pregnancy in previously conducted population-based prevalence studies [29, 30]. In contrast to our study population-based prevalence studies conducted in China preferred pregnant women for fragile X carrier screening. However, some studies also considered both preconception and pregnant women for fragile X carrier screening in different populations of world [31, 32]. Interestingly, in Israel and some parts of USA, pregnant women are not considered for fragile X carrier screening [33, 34]. In addition, fragile X carrier screening is not offered to women at all in many countries as screening may usually pose significant counseling and educational difficulties [35].

Population-based studies have well investigated the prevalence of the fragile X PM carriers among preconception/pregnant women in different populations. The prevalence rates observed were 0.13% in Korean women [18] 0.08 to 0.13% in Chinese women [19, 36], 0.04 to 0.27% % in Australian women [31], 0.88% to 1.3% in Israeli women [29, 37], 0.9% in Spanish women [38]. The overall prevalence rate of fragile X PM carriers was observed 0.7% in this study. Interestingly, no PM carrier was detected in control group women and about 0.5% in RG1 and 0.2% in RG2 women were detected PM carriers thus the observed prevalence in this study was contributed solely by risk groups’ women who had family history of either ID or ASD. Importantly, all risk groups’ women who consulted for preconception care had at least one child or sibling affected with either ID or ASD. Thus, this study and previously reported studies provides evidence of association of positive family history of fragile X-associated disorders with greater risk of preconception carrier women of transmitting the FM to their children. This association has also been found in previous studies [36, 39]. The findings of this study also suggest that FXS could be a reason for the high prevalence of ID and ASD that have been reported in Pakistani population [7, 40, 41].

In this study, we also determined the most significant clinical characteristics among PM carrier women. The risk of FXPOI is increased in PM carrier women and findings of this study supported this fact as PM carriers demonstrated significantly low AMH (< 1 ng/mL) and increased FSH (> 25 IU/L) levels, irregular menstruation, hot splashes and night sweats before age of 40 years compared to non-PM carriers. The observed prevailing FXPOI symptoms act as predictors for early ovarian dysfunction or menopause among PM carriers and the average age for early menopause in FXPOI has already been reported 5 years earlier in PM carrier women than women in general population [42, 43]. Many studies have also noted significantly low AMH and increased FSH levels among PM carriers [42, 44–46]. Similarly, other studies observed irregular, skipped or shorter menstrual cycles and subfertility as prominent features of PM carriers [43, 46]. Hypoestrogenism in PM carrier women may cause variety of clinical conditions [47]. Hypoestrogenism causing osteoporosis has been reported at a high frequency among PM carriers previously [43] as well as in this study, however, it was also found that PM carrier women have no significantly increased risk of osteoporosis.

Previous studies revealed that PM carriers experience significantly antepartum hemorrhage and preeclampsia at least in one pregnancy [13, 47], however, PM carriers were not found at risk for any of these obstetric or perinatal difficulties in the present study. Similarly, in contrast to the study conducted by Wheeler et al. [43], we in this study and Obadia et al. [47] in a previous study examined an increased risk for postpartum depression in PM carrier women who had either one or two children with FXS. Therefore, having more than one child with FXS could be a reason of increased risk of women for postpartum depression, rather than their PM carrier status [47].

Interestingly, in present study all PM carriers showed normal IQ scores compared to their children and siblings with either ID or ASD. The normal IQ scores were also reported in adult PM carriers in previous studies [48, 49]. In contrast, few studies have reported lower IQ scores among PM carriers [50, 51]. Moreover, PM carrier women were at significantly increased risks for various neuropsychiatric features such as anxiety, sleep disturbances, aggression, difficulty in concentrating, hyperreactivity and language issues than non-PM carrier women as these features were present at high frequencies in PM carriers in this study. Previous studies have also reported significantly higher rates of sleep disturbances [52, 53], depression, stress and anxiety [31, 54], language issues [16, 55], attention deficits [16, 48], and memory impairment [56, 57] in PM carriers with or without diagnosis of Fragile X associated disorders compared with controls. Hartley et al. [58] in a study noted predominantly aggression, inattentive, behavior problems and irritability, whereas Chonchaiya et al. [59] observed high prevalence of balance problem, memory loss, dizziness among PM carriers with history of FXD. Prevalence rates of hypertension and migraine were high; however, PM carriers were not found at significantly increased risk for these conditions in this study. In contrast, observed significantly high prevalence of hypertension [60] and migraine [61] in PM carriers with history of FXTAS. Moreover, PM carriers had not an increased risk for diabetes as found in this and previous studies [1, 60].

It is the first study that determined prevalence of fragile X PM alleles in Pakistani women who consulted primary health care centers for preconception care. The PM carrier women had at least one child or sibling affected with either ID or ASD, therefore, this study also provides evidence that FXS could be a reason for the high prevalence of ID and ASD that have been reported in Pakistani population. In addition, the preconception women with *FMR1* PM alleles were found to be at increased risk for FXPOI, postpartum depression and neuropsychiatric disorders. The findings of current study may be used to improve preconception care, direct future screening strategies and educate women about implications of fragile X associated health, reproductive and neuropsychiatric conditions that may greatly help in reducing burden of FXD in our population.

## Data Availability

All the data used to support the findings of this study are included within the article and are available on request from corresponding author.
